# Complications associated with monopolar resectoscopic surgery

**Published:** 2020-05-07

**Authors:** George A Vilos, H Alshankiti, AG Vilos, B Asim Abu-Rafea, A Ternamian

**Affiliations:** The Fertility Clinic, London Health Sciences Centre, Department of Obstetrics and Gynecology, Western University, London, Ontario, Canada;; St. Joseph’s Health Toronto, Department of Obstetrics and Gynecology, University of Toronto.

**Keywords:** Hysteroscopy, uterine perforation, resectoscopic complications, vascular injury, bowel injury, electrosurgery

## Abstract

**Background:**

Resectoscopic injuries to bowel and/or vessels, although rare, can be catastrophic, resulting in significant patient harm including death and can provoke medicolegal litigation.

**Objective:**

To examine indications, preoperative risk factors, perioperative findings and intervention, and clinical outcomes of resectoscopic injuries.

**Materials and methods:**

Eleven cases of resectoscopic complications were reviewed by one author (G.A.V.) for medicolegal purposes. After grouping of the complications, one case for each complication was selected, edited and reconstructed to reflect and highlight all potential complications associated with monopolar resectoscopes (26F, 9-mm) and nonconductive distending medium. Although these cases are reconstructed from actual complications, they do not reflect specific cases of medicolegal opinions and outcomes. Indications for resectoscopic surgery included abnormal uterine bleeding and/or infertility in premenopausal women.

**Results:**

Injuries were associated with uterine perforation resulting in hemorrhage or bowel injury; urinary bladder injury without uterine perforation; and thermal injuries to lower genital tract and dispersive electrode site.

**Conclusions:**

Resectoscopic complications are associated with any one or a combination of trauma during uterine access or intra-operatively, excessive fluid intravasation of distending medium or thermal injuries from applied energy. Uterine perforation in the presence of distorted anatomy (e.g. uterine fibroids) may be considered as a known and accepted complication. Lower genital tract and dispersive electrode site burn occur due to inherent design of monopolar resectoscopes. Appropriate intra- and post-operative intervention minimizes adverse clinical and medicolegal outcomes. Lack of post-operative vigilance and inappropriate delay in investigation and intervention is associated with adverse clinical and, potentially, unfavourable legal outcomes.

**What is new?:**

Reviewing resectoscopic complications raises awareness; provides insight for avoidance, recognition and timely intervention to minimise adverse clinical and medicolegal outcomes.

## Introduction

To perform operative hysteroscopy, surgeons have to access the uterine cavity through the cervix, distend the endometrial cavity with a fluid to visualise and apply energy to affect tissue (ablate, cut, morcellate, coagulate), and to retrieve tissue. Mechanical instruments include scissors or cutting electro- mechanical blades, while coagulation of bleeding and resection or ablation of tissue can be achieved by radio-frequency (RF) electrosurgery or Laser energy. Consequently, most resectoscopic complications are associated with any one or a combination of three incidents: 1) traumatic injury during access such as cervical tears or partial or complete uterine perforation with instruments such as dilators, scissors, electrodes or the hysteroscope/resectoscope; 2) excessive fluid intravasation of distending liquid or gas (room air or generated bubbles), and 3) thermal injuries from applied energy.

Some of these injuries to bowel and/or vessels can be catastrophic, resulting in significant patient harm and even mortality that can provoke medicolegal litigation. Herein, we describe reconstructed cases associated with monopolar resectoscopic surgery encompassing all causes of potential complications listed above and provide insights and recommendations on prevention, recognition and timely intervention for both minor and major complications to minimise or avoid the risk of adverse clinical and medicolegal outcomes.

## Materials and methods

Eleven cases of resectoscopic complications were referred to and were reviewed by one author (G.A.V.) for potential of, or after initiation of litigation. One case for each injury category was selected and reconstructed by all authors to highlight all potential complications associated with operative hysteroscopy using 26F (9 mm) monopolar resectoscopes and nonconductive distending medium. Although these cases represent real complications, they do not necessarily reflect actual cases of medicolegal opinions and outcomes.

All women were premenopausal, ranging from 30 to 46 years of age with a BMI of 23 to 43. Surgery included hysteroscopic endometrial ablation (HEA) with and without myomectomy or polypectomy in women presenting with AUB, dysmenorrhea and/or wishing to enhance or retain fertility. The procedures were performed in both teaching and nonteaching hospitals under general anesthesia using 26F monopolar resectoscopes and 1.5% glycine distending solution. A detailed summary of each example case is provided bellow.

Since we could not possibly obtain individual consent from patients and health care providers, we made these cases essentially fictitious as they are constructed from multiple reviewed cases to highlight specific resectoscopic complications. Therefore, we feel that no institutional review and ethics approval was required.

## Results

### Uterine perforation and vascular injury

#### Resectoscopic myomectomy, uterine perforation, retroperitoneal vascular injury

Two cases of uterine perforation associated with pelvic vessel injury and major intra-abdominal hemorrhage were reviewed as summarised in Table I and one case is described in detail. The frequency of such a complication is extremely rare and it remains unknown. We have not encountered such a complication in over 8000 resectoscopic procedures.

**Table I:** Patient demographics, presentation, surgery, intervention and clinical and outcomes of two vascular injuries associated with hysteroscopic myomectomy. AUB-abnormal uterine bleeding, DIC-disseminated intravascular coagulation.

Case	Age	Parity	BMI	Presentation	Surgery	Uterine perforation	Intervention	Clinical outcome
1	35	0000	23	AUB	Myomectomy	Recognized intra-operatively but not acted upon	Patient decompensated in recovery room in 1 hour. Laparotomy, vessel repair	Good
2	46	0000	-	AUB	Myomectomy and endometrial resection	Not recognized	Patient decompensated on the table. Laparotomy, vessel repair	DIC, multi-organ failure, death

*Case:* A 35-year-old nulliparous woman, BMI 23, presented with abnormal uterine bleeding (AUB). Ultrasound indicated an endometrial mass and she was consented for hysteroscopic polypectomy or myomectomy. The cervix was dilated to 9 mm and hysteroscopy identified a posterior, type 2, submucosal myoma, approximately 4 cm. Using an 8 mm monopolar loop electrode and 120 w ‘cut’ and 80 w ‘coagulation’ power setting, resection of the myoma was initiated. After resecting approximately one third of the myoma, the patient jumped/ jerked during energy application and the loop was momentarily not visualised. A curette passed through the uterine fundus indicating a uterine perforation. The distending medium, glycine deficit was 2355 mL (In-8055 mL, Out-5700 mL). In view of the uterine perforation and the excessive fluid loss, the procedure was terminated. Laparoscopy was considered, but it was felt that the risk of damage to viscera or vessel was low.

In the recovery room, one hour later, the patient appeared pale and diaphoretic. She was hypotensive (BP = 84/30 mmHg), tachycardic and the abdomen was distended. She was resuscitated with 2 units of red blood cells and 1L of saline and she was taken back to the operative room. At laparotomy, following evacuation of large amount of blood, a 1-cm rent in the posterior superior wall of uterus was bleeding and was sutured immediately. A large retroperitoneal hematoma along the left lateral pelvic sidewall was noted and a urologist and vascular surgeon explored the retroperitoneal space and a brisk bleeder from a branch of the internal iliac artery was clipped. The bowel was run and there was no other obvious injury.

Bloodwork indicated Hb-27 g/L, INR-2.1, Na-128. The patient developed disseminated intravascular coagulation (DIC) and she received ten units of packed red blood cells, platelets, and fresh frozen plasma. After care in the intensive care unit (ICU), the patient recovered uneventfully.

*Main lesson from these cases:* The injury was attributed to the uterine perforation and direct vascular laceration with the loop electrode. Uterine perforation during resectoscopic surgery is a known complication and, in the presence of distorted anatomy (e.g. uterine fibroids), it can be an accepted complication.

### Uterine perforation and bowel injury

#### Hysteroscopic myomectomy, rollerball endometrial ablation, uterine perforation, bowel burns

Four cases of uterine perforation associated with bowel injury were reviewed and summarised in Table II. One case is presented in detail. The frequency of such a complication remains unknown. We have not encountered such a complication in over 8000 resectoscopic procedures.

**Table II:** Patient demographics, presentation, surgery, injury, intervention and clinical outcome of 4 bowel injuries during hysteroscopic myomectomy/endometrial ablation. AUB-abnormal uterine bleeding, POD-postoperative day, ICU-intensive care unit, DVT-deep vein thrombosis, DIC-Disseminated intravascular coagulopathy.

Case	Age	Parity	BMI	Presentation	Surgery	Uterine perforation	Intervention	Clinical outcome
3	40	2002	43	AUB	Myomectomy. Rollerball ablation	Recognized	POD #5 Laparotomy, bowel burns	Good
4	44	-	-	AUB	Rollerball ablation	Recognized. Excessive fluid loss	POD #1 Laparotomy, bowel burns	Wound infection. Ventral hernia
5	44	0000	-	AUB, infertility	D&C, Multiple polypectomy	Recognized	POD #4 Laparotomy, bowel burns	Good. ICU care
6	45	-	32	AUB	Myomectomy	Suspected	POD #3 Laparotomy, colon resection	Hartmann, DVT, DIC

Case: A 40-year old woman, P2G2, BMI 43, presented with AUB and dysmenorrhea. Following proposed treatment options, the patient chose hysteroscopic endometrial ablation (HEA). At hysteroscopy, a 3 cm submucosal myoma was noted and it was resected with an 8 mm loop electrode using 80 w of ‘cut’ waveform, followed by rollerball endometrial ablation using 80 w of ‘coag’ power. At the base of the resected myoma, a small fundal uterine perforation was noted and hemostasis was achieved with the rollerball. The fluid deficit was reported as normal.

The patient remained in the post-anesthesia care unit (PACU) for approximately 4 hours because of unusual pain requiring intravenous morphine. The pain persisted at home and on post-operative day (POD) #4, the patient attended the emergency room (ER) of a peripheral hospital after she was awakened with excruciating lower abdominal cramp-like pains. The vitals were: T-37.30C, P-80, BP-120/55. The abdomen was distended and tender with rebound and guarding signs and she was transferred to the original tertiary hospital. The white blood cell count (WBC) was 16 x 10 9 and X-rays indicated free air and large bowel obstruction.

At laparotomy, there was no gross spillage of stool, pus or other large amount of fluid. A perforation of the uterus was noted at the posterior fundus. On running the small bowel, it was found that she had an intramural ‘cautery’ burn area, approximately 2 cm in size, on the mid jejunal area and 10 cm further down there was another superficial serosal ‘cautery’ burn. There were two other burns in the jejunum approximately 10-15 cm distal to the previous perforation. Primary closure of the 3 superficial burns was performed, while a 14 cm length of small bowel including the area of the ‘cautery’ burn-perforation was resected. The patient had an uneventful recovery.

*Main lessons from these cases:* Although the uterine perforation was noted intra-operatively in three cases and suspected in the fourth case, the potential of intra-abdominal injury and exploration of the abdomen was not considered. Furthermore, vigilant observation in the PACU or following admission to the hospital of the index case was not exercised; instead the patient was discharged in spite of excessive postoperative pain in the PACU. Expert opinion was critical in all four cases for delayed intervention in the phase of recognised or suspected uterine perforation but not acted upon accordingly.

### Excessive intravasation of distending medium and generated bubbles

Excessive intravasation of nonconducting solutions (amino acids, sugars, free water) can result in significant hyponatremia with serious adverse sequelae. Likely due to strict vigilance of fluid monitoring manually or using automated systems, we have not encountered such a complication in our practice. We have however, reported on the mechanism of a patient’s death from acute hyponatremia associated with excessive water infusion following a hysterectomy (Vilos et al., 2019a). In addition, we have reported on the frequency (5 cases among 5707) and mechanism of venous gas embolism during hysteroscopic endometrial ablation (Vilos et al., 2019b)

### Electrosurgical Burns

#### Repeat hysteroscopic rollerball endometrial ablation, burn to bladder without uterine perforation:

*Case:* A 42-year-old woman, G1P1, with one previous Caesarian section, presented with persistent AUB two years after thermal balloon endometrial ablation (TBEA). After discussing treatment options including a hysterectomy, she was offered repeat hysteroscopic endometrial ablation using a rollerball. Cervical Papanicolaou smear and endometrial biopsy were normal, and pelvic ultrasound indicated a 7.9 x 3.5 x 4.2 cm anteverted uterus. Pre-operatively, she was treated with a single injection of Leuprolide acetate (3.75 mg, Abbvie, St. Laurent, Quebec).

Intra-operatively, the cervix was dilated up to 9 mm. At hysteroscopy, the uterine cavity appeared bicornuate, thought to be a result of the previous TBEA. Using a power setting of 80 w and a 5-mm rollerball, all visible endometrium was ablated including one area of deep endometrial pocket (isthmocele) in the lower anterior cervical/ uterine junction. There was no evidence of uterine perforation.

Five days postoperatively, she experienced dysuria, nausea, vomiting and low-grade fever. Urinalysis was positive for red and white blood cells and although there was no bacterial growth in the urine culture, she was treated with oral antimicrobials. Her symptoms of urinary frequency, urgency and haematuria persisted and, in addition, she experienced vaginal bleeding with passing clots. Cystoscopy showed thermal injury above the trigone at the postero-lateral aspect of the right side of the bladder and CT scan showed focal thickening of the same area described in cystoscopy. She was treated conservatively with oral antibiotics and analgesics and recovered without further intervention.

*Main lesson from this case:* It was proposed that the most likely predisposing factor for the thermal injury to the base of the bladder was the distorted anatomy of the uterine cavity which, most likely, was related to her previous TBEA and a thin myometrial area at the previous Caesarean section scar (isthmocele). Under such conditions, complications may be unpredictable and frequently unavoidable.

### Lower Genital Tract Burns

#### Hysteroscopic rollerball endometrial ablation, burns to the lower genital tract

Three cases of women presenting with AUB, aged 34 to 45 years, underwent hysteroscopic endometrial ablation using a rollerball and one case is presented in detail.

*Case:* A 42-year-old woman, G2P2, presented with AUB and dysmenorrhea. She was offered medical therapies, but she chose hysteroscopic endometrial ablation after discussing the risks and benefits in detail. She was started on danazol, 200 mg (Sanofi-Aventis, Laval, QC), twice daily for six weeks to thin the endometrial lining.

**Figure 1:**
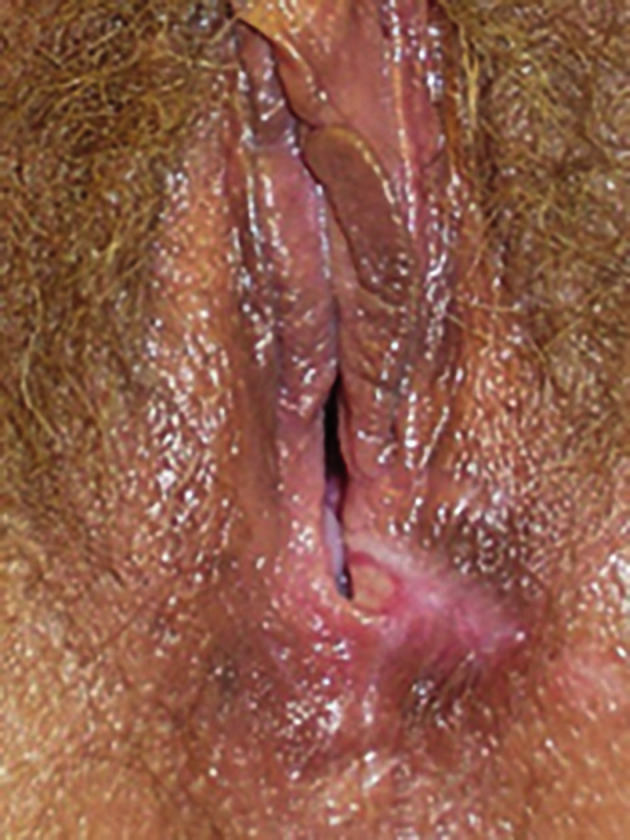
— Thermal burn of the vaginal introitus associated with endometrial ablation using a 26F monopolar resectoscope and a 5 mm rollerball.

Using a monopolar resectoscope, endometrial ablation was performed with a 5 mm rollerball using 85 w ‘coag’ power and 1.5% glycine distending medium. At the end of the procedure, a burned area on the posterior vaginal wall and perineum was noted. It was white without epithelial sloughing (Figure 1). The burn was treated with silver sulfadiazine cream (flamazine, Bowers Medical Supplies, Delta, BC) and vaginal packing and healed with minimal scar. The patient alleged chronic dyspareunia. Electrosurgical lower genital tract burns are quite rare. We have not encountered a single case in over 8000 resectoscopic procedures.

*Main lessons from these cases:* Engineering reports from the hospital and/or the manufacturer of the resectoscope stated that there was no fault with any of the instruments used in these cases. However, it has been demonstrated that monopolar electrosurgery is associated with capacitive coupled currents induced on the external sheath of the resectoscope, which together with possible stray currents from insulation defects of the electrode, can cause genital tract burns, due to inherent design of all monopolar resectoscopes.

### Dispersive Electrode Site Burns

#### Hysteroscopic rollerball endometrial ablation, dispersive electrode site burn

**Figure 2:**
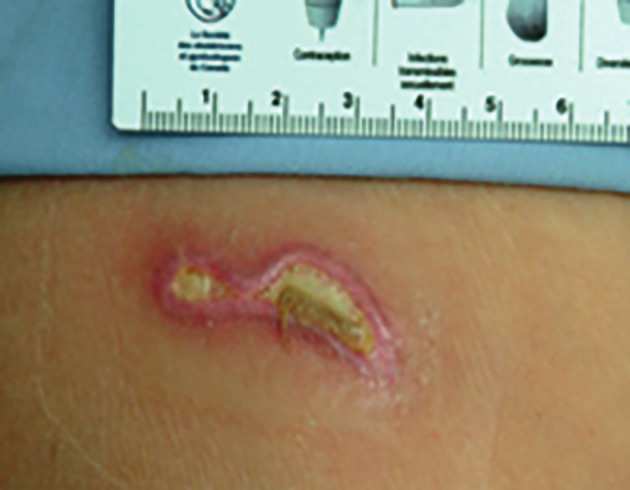
— Dispersive electrode burn associated with monopolar resectoscopic surgery using a 26F resectoscope (the picture is taken one week after the incident).

*Case:* A 42-year-old woman presented with AUB. After discussing treatment options, she elected to undergo HEA. Using a 26F monopolar resectoscope, 1.5% glycine distending solution and a 5 mm rollerball electrode at 120 w of power, provided by an electrosurgical unit (ESU) equipped with a Contact Quality control Monitoring System (CQMS), the endometrial ablation was completed uneventfully. After detaching the split dispersive electrode, a second degree burn with blistering of the skin was noted over its attachment. A picture taken one week after the initial burn is shown in Figure 2. The burn was treated with silver sulfadiazine cream (flamazine, Bowers Medical Supplies, Delta, BC) by a plastic surgeon and healed uneventfully after approximately 2 months. Dispersive electrode site burns are quite rare. We have encountered only one case in a little over 8000 resectoscopic procedures.

Main lesson from this case: Following such burns, the manufacturers of the dispersive electrodes assume responsibility in accordance with their indemnification clause stating: “Guarantee Indemnification. We offer the broadest split-pad indemnification in the industry. Use the 3M split- pad with any brand electrosurgical generator equipped with a CQMS [Contact Quality control Monitoring System, REM TM or NESSY TM style) safety system, and 3M will indemnify your hospital and its employees, medical and professional staff”. A similar Patient Safety Guaranteed clause has been maintained by Valleylab. “Valleylab’s REM [Return Electrode Monitoring] Hold Harmless Agreement indemnifies the hospital, surgeon, and OR staff from liability, if a pad-site burn occurs while using Valleylab equipment, a REM-equipped electrosurgical generator and REM patient return electrode”. (REM TM , Valleylab, a division of Tyco, Boulder, CO, USA) and Neutral Electrode Safety System (NESSY TM, ERBE, Tubingen, Germany).

## Discussion

### 

These resectoscopic complications, personal knowledge and experience, and review of pertinent literature, allows us to make several observations and provide summary statements and conclusions, as well as suggest future directions in clinical practice to minimise risk of injury and potential litigation associated with hysteroscopy and resectoscopic surgery.

*Uterine perforation during hysteroscopic surgery:* The first observation is that major hysteroscopic complications are associated with uterine perforation and injury to intra-abdominal tissues and/or organs. Perforation of the uterus can occur with a uterine sound, cervical dilators, mechanical grasping tools, or the hysteroscopic/resectoscopic system.

The rate of uterine perforation during resectoscopic surgery has been estimated at <1%. In a prospective multicentre trial including 13,600 cases, the uterine perforation rate during diagnostic vs. operative hysteroscopy was 0.13% vs. 0.76%, respectively (Jansen et al., 2000). In a 1997 National survey of 10,686 hysteroscopic endometrial resections performed by 690 physicians (1-222 cases/doctor) from April 1993 to October 1994 in the UK, the rate of perforation was 2.47% (88 cases), three of which (0.08%) were associated with visceral burns (Overton et al., 1997).

### Avoidance or minimising the risk of uterine perforation

*Intra-operative considerations:* Prior to the insertion of any instrument into the uterine cavity through the cervix, it is imperative that the surgeon performs a bimanual pelvic examination to determine the size, shape, consistency and, especially, the position of the uterine body in relationship to the cervix (anteverted, retroverted, anteflexed, retroflexed, mid position etc.). The first dilator then should follow the appropriate direction of the cervical canal and advanced into the uterine cavity with minimal or no resistance.

If excessive resistance is encountered, one may use a small diameter (<5 mm) hysteroscope to negotiate the cervical canal and enter the uterine cavity under direct visualisation. In the absence of a small diameter hysteroscope, the cervix can be ‘softened’ with injection of 5 to 10 mL of a local anesthetic with or without a vasopressor into the paracervical tissue.

*Pre-operative cervical ripening:* If cervical stenosis is suspected prior to surgery, one should consider ripening the cervix with a prostaglandin. A 2015 Cochrane review of 19 RCTs with a total of 1870 participants reported on comparing misoprostol with no treatment or placebo, dinoprostone or osmotic dilators. The authors reported that misoprostol was more effective for cervical dilatation than placebo or no intervention, with fewer women requiring mechanical dilatation. However, there was no evidence of a difference between the groups in rates of uterine perforation (low quality evidence). The authors concluded that there is moderate quality evidence that use of misoprostol for preoperative ripening of the cervix before operative hysteroscopy is more effective than placebo or no treatment and is associated with fewer intraoperative complications such as lacerations and false tracks. However, misoprostol is associated with more side effects, including preoperative pain and vaginal bleeding (Al-Fozan et al., 2015).

*Pre-operative endometrial thinning:* Intuitively, a thin non-polypoid endometrium with reduced vascularity should improve visualisation and potentially minimise the risk of intra-operative uterine perforation. A 2013 Cochrane review reported on 20 studies with 1969 women comparing gonadotropin hormone releasing agonist (GnRHa), danazol and progestogens versus placebo or no treatment; GnRHa versus danazol, progestogens, GnRH antagonists or dilatation & curettage; and danazol versus progestogens. When compared with no treatment, GnRHa used before hysteroscopic resection were associated with a slightly shorter duration of surgery and greater ease of surgery. However, although the use of GnRHa produced slightly more consistent endometrial thinning, it had no effect on intraoperative complication rates. The authors stated that low-quality evidence suggests that endometrial thinning with GnRHa and danazol before hysteroscopic surgery improves operating conditions and short-term postoperative outcomes (Tan et al., 2013).

*Uterine perforation and vascular injury:* In cases of uterine perforation and major vascular injury, the injury declares itself early, prompting timely intervention. However, these injuries can be catastrophic as in our second case, where hemorrhage was severe enough that although intervention and repair was timely, the patient died from disseminated intravascular coagulation (DIC) and multiple organ failure in spite of multiple transfusions of blood and blood products.

*Uterine perforation and bowel injury:* Clinical practice guidelines recommend that if perforation occurs with mechanical instruments, in the absence of obvious bleeding and no suspicion of organ injury, the patient can be treated expectantly. Laparoscopy or laparotomy should be reserved for those circumstances where organ (bowel, bladder, etc.) injury is suspected, where there appears to be a large uterine defect, or in the presence of excessive and/or persistent bleeding. However, if the perforation occurs with an activated electrode or the surgeon is uncertain of whether the electrode was activated or not at the time of perforation, then one has to assume that there has been a thermal organ injury, until proven otherwise. Under these circumstances, exploratory laparoscopy or laparotomy is recommended (Laberge et al., 2015).

Alternatively, following a recognised or suspected uterine perforation, a watchful waiting approach may be acceptable provided that the patient is monitored and observed vigilantly and the physician intervenes at the earliest sign of impeding trouble declared by increasing pain, and/or changes in the patient’s vital signs. (Temperature, blood pressure, heart rate, respiratory rate, urine output, abdominal signs, etc.).

In our cases, although the uterine perforation was recognised in three cases and suspected in one case, exploration of the abdomen was not considered, resulting in delayed investigation and intervention and adverse clinical outcomes. Therefore, uterine perforation is a well-known and accepted complication of any hysteroscopic surgery. Uterine perforation however, when suspected or recognised intra-operatively, should be dealt with in accordance with established clinical practice guidelines.

*Postoperative pain:* Another important clinical caveat from these cases is that postoperative signs and symptoms should be monitored vigilantly and acted upon in a timely fashion. In general, hysteroscopic surgery is associated with minimal intra- and post-operative pain and patients are expected to be discharged approximately two hours after surgery. Furthermore, a general rule of surgery is that post-operative pain should improve with time, and that persistent or increasing pain is a symptom of possible organ injury and/or occult bleeding that must not be ignored. In the case of bowel injury, postoperative pain was excessive in the PACU and at home after discharge but it was not investigated appropriately and acted upon in a timely fashion.

### Electrosurgical thermal resectoscopic surgery

#### 

An additional important lesson from these cases is that visceral thermal injuries can occur with the active electrode (rollerball/loop, etc.) with or without uterine perforation; genital tract burns can occur due to capacitive coupling or insulation failure associated with all monopolar resectoscopes; and, dispersive electrode burns occur due to the design of the dispersive electrodes and/or misunderstanding of the principles of electrosurgery.

#### Thermal injury with the active electrode without perforation:

In this case, the most likely cause of thermal injury to the base of the bladder with the rollerball was a thin endo-myometrial wall at a previous Caesarean section site. Also, following thermal balloon endometrial ablation (TBEA), the uterine cavity is distorted in up to 75% of cases (Garcia-Erdeljan and Vilos, 2010). We have also reported from another study that complications occurred in 9.3% of repeat ablations compared with 2.1% of primary ablations (p = 0.006) and concluded that repeat endometrial ablation has a significantly higher rate of perioperative complications than primary ablation (MacLean et al., 2002). However, in a later study involving repeat ablation in 183 women using exclusively the loop electrode to resect rather than rollerball residual endometrium, uterine perforation occurred in only 2 (1.1%) cases (Yeung et al., 2015).

#### Genital tract burns during hysteroscopic rollerball endometrial ablation:

The first three Canadian cases of thermal burns along the genital tract associated with rollerball endometrial ablation were first published by Vilos et al. (1997). Subsequently, an additional 10 cases from the United States were collected and published in 2000 (Vilos et al., 2000a). All of the above cases were involved in litigation against the gynaecologist, the hospital and the manufacturers of the resectoscopes used.

Experiments in the lab using animal tissue identified potential mechanisms for such injury and reproduced such burns using similar equipment to the ones used by surgeons (Vilos et al., 2000b; Munro, 2004). The mechanism responsible for such burns may be a combination of stray electrical currents induced by intact electrodes and/or resectoscopes (capacitive coupled current), defective insulation of the electrodes and/or the resectoscope system (insulation failure) and, possibly, ionic currents generated by blood mixing with the non-conductive solutions used to irrigate and distend the uterus (Vilos et al., 2006).

#### Capacitive coupling of monopolar resectoscopes:

The physics of all monopolar resectoscopes’ arrangement, unintentionally allows for an electrical capacitor system. Detailed discussion of the mechanism of capacitive coupled currents has been described previously (Vilos et al., 2000b; Munro, 2004; Vilos et al., 2006).

Capacitive coupled currents are eliminated by so- called, bipolar resectoscopes. In such resectoscopes, the arrangement allows for the current on the active and return electrodes to travel in close proximity to one another (the two insulated cables are adhered to each other). Under this configuration, the currents travel in opposite directions and the corona discharge (capacitance currents) generated by each cable cancel each other out. In addition, bipolar resectoscopes operate at significantly lower voltage than monopolar electrodes. Based on the above physics, we investigated and published on the first coaxial bipolar electrodes that cut, desiccate (dry up) and vaporise intrauterine lesions in a saline environment (Vilos, 1999).

*Insulation Failure of resectoscopes:* Resectoscopic electrodes (rollerball, bar, loop, etc.) are insulated throughout their length, except at the distal rollerball or loop and approximately one cm at the proximal end which is inserted into the block of the working element of the resectoscope and subsequently connects to the cable coming from the generator. Insulation defects which may not be visually perceptible along the shaft of the electrode may cause arcing (spark, short) or direct coupling to the telescope, thus electrifying the entire resectoscope.

Munro, from in-vitro studies determined that proximally located electrode insulation defects allowed induction of most of the generator’s output to the external sheath when high-voltage modulated outputs (coag setting) were used, and the risk varied somewhat with the model of ESU used. He concluded that in the presence of proximal electrode defects, high-voltage [coag] currents may contribute to thermal injury to the lower genital tract during RF resectoscopic surgery (Munro, 2003).

*Ionic Currents:* An additional source of stray current, we termed ionic current, was identified in- vivo measurements and published by our group in 2006. These ionic currents emerged when blood was mixing with the non-conductive solution (glycine) and some of these surges were of sufficient amplitude and duration to exceed the calculated heat factor (Heat Factor = current x current x duration of activation, HF = I 2 x t) and potentially cause vaginal burns (Vilos et al., 2006.

*Bipolar Resectoscopes:* When using bipolar resectoscopes the capacitance currents cancel each other out. In addition, ionic currents do not pose a problem since bipolar resectoscopes can only function in conductive solutions as first described by Vilos (1999). Since then, most manufacturers have developed bipolar resectoscopes marketing them in conjunction with their monopolar resectoscopes. Interestingly, although some manufacturers are fully aware of the monopolar resectoscopes being associated with perineal burns, they have not recalled them from the market and continue to sell and promote their use.

### Dispersive Electrode Burns

Several cases of dispersive electrode burns have been reported. In one case, a specially designed vaporising electrode was used to vaporise a submucosal leiomyoma and the power was incrementally increased up to 300 w using continuous waveform (cut) current and a Valleylab ESU with REM system. No ESU alarms occurred and at completion of the procedure, a deep second to-third-degree burn at the site of the dispersive electrode was noted which required skin grafting (Rader & Vilos, 1999).

Mechanism of Dispersive Electrode Site Burns: The heat generated at the electrode-tissue interface (Heat Factor) is directly proportional to the square of the current delivered times the duration of its application (HF= I 2 x t). Therefore, contributing factors to a dispersive electrode burn are:

1. Increased current. (In the cut mode, when current is delivered 100% of the time);
2. Prolonged activation of the ESU;
3. Using electrolytic solution with monopolar energy which dissipates the energy throughout the solution and uterine tissue with no apparent effect on the tissue.

### Future directions

*Hysteroscopic versus nonhysteroscopic endometrial ablation:* A 2019 Cochrane review stated that approaches to endometrial ablation have evolved from first-generation (also referred to as hysteroscopic techniques) to newer second- and third-generation approaches (also referred to as nonhysteroscopic, automated or global ablation). Current evidence suggests that compared to first-generation techniques, second-generation approaches are of equivalent efficacy for heavy menstrual bleeding, with comparable clinical outcomes. However, second-generation techniques are associated with shorter operating times and are performed more under local rather than general anaesthesia. It is uncertain whether perforation rates differed between second- and first-generation techniques (Bolfill Rodriguez et al., 2019).

Hysteroscopic tissue removal systems: A 2018 systematic review and meta-analysis reported on 5 studies including 498 patients comparing mechanical hysteroscopic tissue removal systems (Truclear ® , Myosure ® or IBS ® ) versus conventional bipolar and monopolar resectoscopy for the treatment of polyp and myoma removal. The study reported that hysteroscopic tissue removal systems showed a significantly higher success rate of complete endometrial pathology removal (P=0.002) and a significantly shorter operation time for polyp removal (P<0.0001) compared to conventional resectoscopy. No significant differences, in terms of complications rate, were found (P=0.09) but the fluid deficit was significantly higher in the tissue removal system group, compared to conventional resectoscopy (P=0.02) (Yin et al., 2018).

## Summary statements and conclusions

The main findings of this manuscript have important clinical and medicolegal implications including the following:

*Inherent risk:* Some of the resectoscopic complications are due to inherent risks of the procedure and/or instrumentation. Under such circumstances, harmful incidents can occur despite the surgery being performed by qualified staff using appropriate knowledge, experience, technique, and available equipment.

*Uterine perforation:* Uterine perforation during resectoscopic surgery is a known complication and, in the presence of distorted anatomy (e.g. uterine fibroids), it can be an inherent risk of the procedure.

*Uterine perforation and vessel injury:* Major bleeding associated with vascular injury usually declares itself immediately after uterine perforation prompting immediate intervention which may minimize adverse clinical and medicolegal outcomes.

*Uterine perforation and bowel injury:* Uterine perforation associated with bowel injury should be dealt with in accordance with recommendations of clinical practice guidelines as stated below.

Intra-operatively:

- During hysteroscopic surgery, if uterine perforation occurs with mechanical instruments, in the absence of obvious bleeding and no suspicion of organ injury, the patient can be treated expectantly;
- Laparoscopy should be reserved for those circumstances where organ (bowel, bladder, etc.) injury is suspected, where there appears to be a large uterine defect, or in the presence of heavy bleeding;
- If perforation occurs with an activated electrode, or the surgeon is uncertain of whether the electrode was activated or not at the time of perforation, exploratory laparoscopy or laparotomy is recommended.

Post-operatively:

- Following hysteroscopic surgery, pain should be getting progressively better and patients should be improving with time;
- Strict vigilance and appropriate investigation and intervention is indicated in the presence of persistent or escalating pain or other signs and symptoms indicative of intra-abdominal issues.

*Visceral burns without uterine perforation:* A thin myometrial wall from previous uterine surgery (e.g. thin Caesarean section scar, isthmocele) or partial perforation may allow sufficient heat energy transfer to burn adjacent organs(s).

*Lower genital tract burns associated with monopolar resectoscopes:* Stray currents (capacitive coupled, insulation failure, ionic currents) generated on the external sheath of monopolar resectoscopes are inherent due to the utilisation of high frequency currents (>500, 000 Hz, RF).

Recommendation to avoid RF burns:

- Use bipolar resectoscopes;
- Leave weighted or any metal speculum in the posterior vagina to dissipate stray currents throughout the vagina and back to the dispersive electrode safely.

*Dispersive electrode site burns:* Heat generated (HF, heat factor) at the dispersive electrode/tissue interface is directly proportional to the square of the current used, times the duration of the applied current. (HF = I^2^ x t)

Recommendation to minimize RF dispersive electrode burns:

- Use lowest possible power to achieve desired surgical effect;
- Interrupt application of current to minimise excessive heat on dispersive electrode;
- Use capacitive coupled return electrodes (e.g., Megadyne pads, Megadyne, Draper, UT).
